# Revealing Differential Expression of Phytohormones in Sorghum in Response to Aphid Attack Using the Metabolomics Approach

**DOI:** 10.3390/ijms232213782

**Published:** 2022-11-09

**Authors:** Jian Huang, Kumar Shrestha, Yinghua Huang

**Affiliations:** 1 Department of Plant and Soil Sciences, Oklahoma State University, Stillwater, OK 74078, USA; 2 Department of Plant Biology, Ecology and Evolution, Oklahoma State University, Stillwater, OK 74078, USA; 3 USDA-ARS Plant Science Research Laboratory, 1301N Western Road, Stillwater, OK 74075, USA

**Keywords:** aphid, host plant defense, insect resistance, jasmonic acid, metabolomics, phytohormone, plant and insect interaction, salicylic acid, sorghum

## Abstract

Sorghum (*Sorghum bicolor*) is an important multipurpose crop grown worldwide, but like many other crops, it is often threatened by insect pests. Sugarcane aphid (SCA, *Melanaphis sacchari* Zehntner), for example, is one of the most severe pests in sorghum, which causes plant damage and yield loss. The main objective of this study was to assess the effect of phytohormones on host plant resistance to aphid attack. Two sorghum genotypes, BTx623 (susceptible) and Tx2783 (resistant), were selected for a comparative analysis of differential expression of a group of phytohormones in response to aphid infestation. The quantification of phytohormones through LC-MS demonstrated higher levels of jasmonic acid (JA), salicylic acid (SA), abscisic acid (ABA), and auxins in the resistant genotype infested with SCA. The PCA plot supports the strong differential responses between resistant and susceptible genotypes, indicating a positive correlation between JA and ABA and a negative correlation between SA and auxins. Similarly, RT-PCR results of the phytohormones-related marker genes showed higher expression in the resistant genotype compared to the susceptible one. Furthermore, to corroborate the role of phytohormones in plant defense, the susceptible genotype was treated with SA, JA, and ABA. The exogenous application of SA and JA + ABA significantly reduced plant mortality, aphid number, and damage in the susceptible genotype, suggesting a strong correlation between phytohormones and plant survival. Our findings indicate that phytohormones play positive roles in plant defense against aphids and provide new insights into the molecular mechanisms operating in plants for self-protection. These findings could also stimulate further research into the mystery about the regulation of phytohormone production during plant interaction with aphids.

## 1. Introduction

Sorghum (*Sorghum bicolor* L. Moench) is a warm season, multipurpose annual crop grown worldwide, providing food, feed, fiber, and fuels (a source of biofuels) [1]. Sorghums are highly productive crops and possess the C4 photosynthetic pathway allowing very efficient assimilation of carbon at high temperatures, contributing to higher productivity. They are very drought tolerant and have high water-use efficiency, which makes it highly successful. Sorghum performs relatively well under water scarcity and elevated temperature conditions compared with the major cereal crops wheat, rice, and maize [2]. Therefore, sorghum is considered as a climate resilient crop for food and nutrition security [3]. However, like many other crop species, sorghum is not immune to damaging biotic stresses such as plant diseases and insect pests, which cause severe plant damage and significant yield loss [4].

In 2013, a new aphid pest, the sugarcane aphid (SCA, *Melanaphis sacchari* Zehntner), was reported to be damaging sorghum along the Texas Gulf Coast and Louisiana [5]. SCA survived the 2013 winter in Texas and spread throughout much of Texas and 12 other southern states during the spring and summer of 2014. In 2015, SCA spread through Texas, Oklahoma, and Kansas while progressing eastwardly, infesting over 17 states covering 90 percent of the US sorghum acreage [6]. The sugarcane aphid is currently one of the most important pests of grain and forage sorghum in most of the sorghum producing areas. Sugarcane (*Saccharum officinarum* L.) and sorghum are important cultivated hosts, and the sugarcane aphid is one of the devastating economic pests in not only North America but also in Asia, Africa, Australia, and South America [4].

Sustainable production of sorghum crops in the U.S. and elsewhere is dependent on the control of those damaging pests such as the sugarcane aphid. Use of genetically pest-resistant cultivars and hybrids in an integrated pest management program are the most economical and environmentally sound methods to reduce the negative economic impact of these important pests. For this purpose, we recently conducted research on the evaluation of sorghum germplasm and identified genetic resources with excellent resistance to sugarcane aphids [6,7]. Simultaneously, we have been conducting experiments to understand the genetics of aphid resistance in the sorghum plant [8,9,10]. Gene expression profiling offers new insights and tremendous promise for dissecting the host defense mechanisms and regulatory networks that control biological processes. One of these research efforts is on the assessment of phytohormones’ role in host plant resistance against sugarcane aphids [11]. Phytohormones are among the most essential growth regulators and are known for having a noticeable impact on plant metabolism. Furthermore, phytohormones play a critical role in nearly every aspect of plant biology from regulating developmental processes and signaling networks involved in plant responses to a wide range of biotic and abiotic stresses [12]. These multifunctional hormones are involved in plant development and defense systems. In spite of the fact that plant hormones are produced in very low amounts, they regulate many external and internal stimuli to improve the economic yield of crop plants [13,14,15]. 

**Figure 1:**
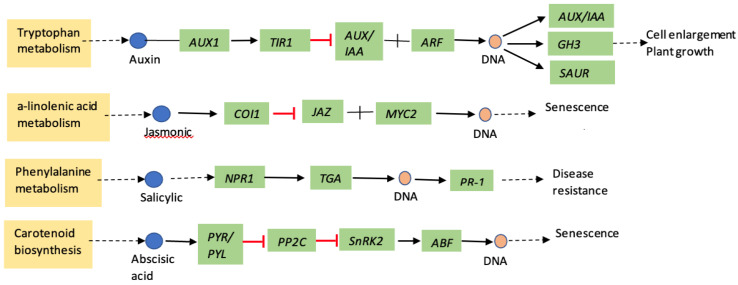
Schematic diagram of major plant hormone signal transduction pathway for sorghum (adapted from KEGG, yellow highlights are pathway name, dotted arrow represents multiple enzymatic steps, green highlights are genes in respective pathways). *AUX—auxin influx carrier; TIR1—transport inhibitor response1; AUX/IAA—auxin/indole-3-acetic acid; ARF—auxin response factor; GH3—auxin responsive Gretchen hagen3; SAUR—small auxin upregulated RNA; COI1—coronatine-insensitive protein 1; JAZ—jasmonate ZIM-domain; MYC2—transcription factor MYC2; NPR1—regulatory protein NPR1; TGA—transcription factor TGA; PR-1—pathogenesis related-1; PYR/PYL—abscisic acid receptor PYR/PYL family; PP2C—protein phosophatase 2C; snRK2—serine/threonine-protein kinase; ABF—ABA responsive element biding factor*.

Progress has been made in identifying the main components and understanding the role of salicylic acid (SA), jasmonic acid (JA), and ethylene (ET) in plant responses to insect attack. Aphid feeding on the host plants are known to induce several plant defense signaling pathways [16]. Many of the plant defense mechanisms involve these signaling molecules: SA, ET, JA, and abscisic acid (ABA). These signaling molecules are induced in a distinct pattern (Figure 1) in host plants in response to attack by insects and plant pathogens [17]. Salicylic acid is one of the predominant phytohormones, which regulates plant responses to pathogens and phloem-feeding insects such as aphids [18,19,20]. In contrast, jasmonic acid often responds to chewing herbivores and wounding [21,22,23]. Similarly, during wounding or insect feeding, ABA acts synergistically with the JA response pathway, while it antagonizes the ET pathway [24,25]. ABA negatively affects SA signaling and promotes the JA responsive pathway, thus affecting SA–JA crosstalk [26]. Importantly, cross-talks between these hormonal signal transduction pathways are thought to enable fine-tuning of plant responses to herbivore and pathogen attack [27]. 

This study was based on the hypothesis that phytohormones are signaling molecules that regulate the crucial responses of host plants to aphid attack. Thus, experiments were conducted for a comparative analysis of differential expression of seven important hormones between two contrasting genotypes (Tx2783 and BTx623) using the metabolomics approach. The results generated from these experiments indicate that both aphid-induced upregulation of phytohormone genes and exogenously applied hormones have an important impact on sorghum plants to cope with aphid attack. These findings provide new insight into a better understanding of the molecular mechanism of host plant defense against aphids and other phloem-sucking insects.

## 2. Results

### 2.1. Differential Response to Aphid between Resistant and Susceptible Genotypes

**Figure 2:**
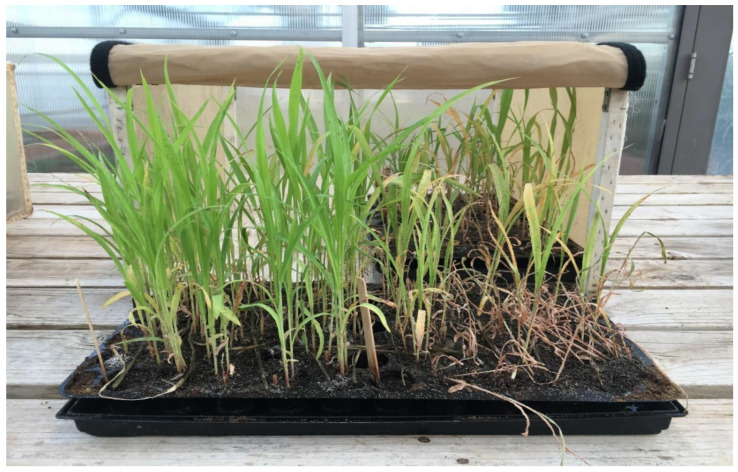
Phenotypes of two sorghum genotypes, Tx2783 (resistant, **left**) and BTx623 (susceptible, **right**) were photographed after co-cultivation with sugarcane aphids for 12 days.

Two genotypes, Tx2783 and BTx623, contrast with each other in response to sugarcane aphid (SCA) infestation. During aphid infestation, Tx2783 showed adverse effect on aphid development and fecundity in comparison to the susceptible genotype (BTx623). The number of aphids per plant was counted and the rate of aphid regeneration was significantly reduced on Tx2783 in comparison to BTx623 (Table 1). Additionally, it appeared that sugarcane aphid caused no visible damage to Tx2783 compared to the severe plant damage in BTx623 (Figure 2). The scores of plant damage on Tx2783 was 0 up to 6- days post infestation (dpi), and slowly increased to 3 at 15-dpi where all BTx623 were dead (Table 1). Based on these results, we conclude that Tx2783 is resistant to SCA while BTx623 is susceptible.

### 2.2. Phytohormone Profiles in Sorghum Seedlings during Plant-Aphid Interaction

**Figure 3:**
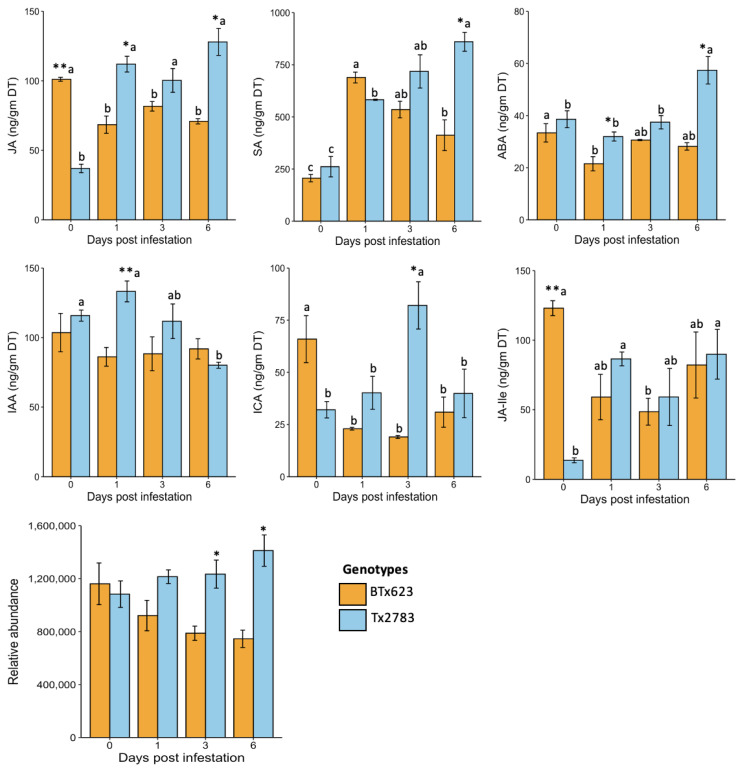
Constitutive levels of seven phytohormones in resistant (Tx2783) and susceptible (BTx623) genotypes during SCA infestation at different time points. The asterisk (*) at the top of each bar represents the significant difference between the genotype at same time points (* *p* < 0.05, ** *p* < 0.01; *t*-test). The letters at the top of each bar represent significant difference between time points within a same genotype (*p* < 0.05; one-way ANOVA). (Error bars represent the mean ± SE (*n* = 3).

To assess the role of phytohormones in sorghum resistance to SCA, we quantified the levels of seven important phytohormones in experimental plants, including JA, SA, ABA, jasmonic acid-isolecucine (JA-Ile), indole-3-carboxylic acid (ICA), indole-3-acetic acid (IAA), and 12-oxo-phytodienoic acid (OPDA). The phytohormones were measured in resistant (Tx2783) and susceptible (BTx623) genotypes at 0-, 1-, 3-, and 6-dpi, respectively. The results in Figure 3 indicate that JA, SA, ABA, IAA, ICA, and OPDA were highly induced in the resistant genotype in comparison to the susceptible one. The constitutive level of JA in BTx623 at the control (0-dpi) time was significantly higher than the Tx2783 and other aphid-infested samples of the same genotype at different time points. In contrast, Tx2783 had a relatively low level of JA at 0-dpi and the levels of JA were significantly higher at 1-, 3-, and 6-dpi. The JA amount was significantly higher in Tx2783 at 1- and 6-dpi compared to BTx623. A similar response was shown by JA-Ile, a biologically active form of JA, except there was no significant difference between BTx623 and Tx2783 during aphid infested time points. The OPDA, a precursor of JA biosynthesis, showed no significant difference between the control (0-dpi) and the aphid-infested samples in either genotype. However, significant difference was observed between BTx623 and Tx2783 during 3- and 6-dpi, where higher amounts of OPDA were noted in the aphid-infested resistant line.

The important defense phytohormone SA had a low constitutive level (0- dpi) in both BTx623 and Tx2783 but was significantly higher in all aphid-infested time points in comparison to the control (0-dpi). The level of SA in both genotypes was similar at all time points except at 6-dpi, where the SA level was significantly higher in Tx2783. Similarly, the expression levels of ABA showed no significant difference at 0-dpi between genotypes but showed significantly higher upregulation in Tx2783 at 1- and 6-dpi. The significant difference in ABA levels was observed between different time points in both genotypes, where there was a slightly higher level of ABA in BTx623 at 0-dpi in comparison to aphid-infested 1-dpi. Conversely, there were no significant changes in the ABA level of Tx2783 at 0-, 1-, and 3-dpi, but only at 6-dpi did it show a significant change. Besides stress-related phytohormones, the growth hormones such as IAA and ICA also showed significant difference between genotypes and at various time points during plant-aphid co-cultivation. The IAA level was significantly higher in Tx2783 at 0-, 1-, and 3-dpi in comparison to 6-dpi, while there was no difference in the susceptible genotype at any time points. Similarly, no significant difference was noted between genotypes except at 1-dpi where Tx2783 showed a higher level of IAA. The constitutive level of ICA in BTx623 at 0-dpi was significantly higher in comparison to the other time points following infestation. Conversely, Tx2783 had a significantly higher level of ICA at 3-dpi in comparison to the other time points. Overall, higher levels of phytohormones were found in the resistant genotype following SCA infestation. 

### 2.3. PCA Indicated the Correlation between Phytohormonal Level and Plant Resistance

**Figure 4:**
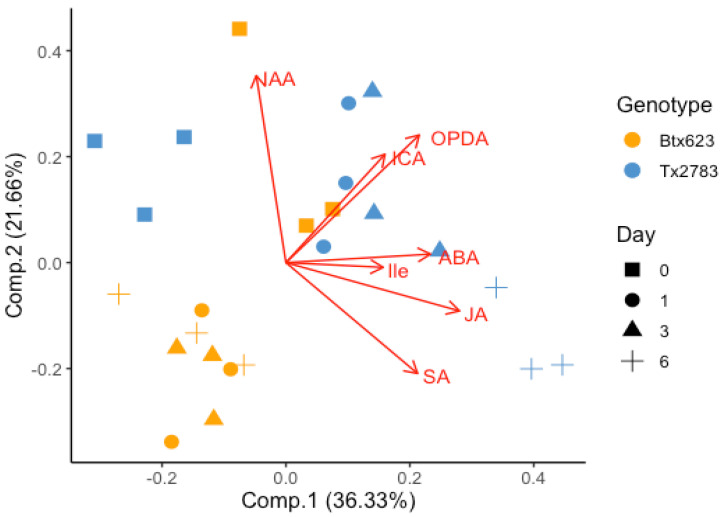
Principal component analysis (PCA) of phytohormones data collected from two sorghum genotypes (Tx2783 and BTx623) at different time points (0-, 1-, 3-, and 6-dpi) infested with sugarcane aphid.

Principal component analysis (PCA) was performed to see the impact of SCA infestation on the phytohormone levels at different time points. In PCA analysis (Figure 4), the component 1 and 2 explained 58% of variability. The first principal component accounting for 36.33% of the variance indicates a strong differential response between the two genotypes and the same for control vs. aphid-infested samples at the hormonal level. Similarly, the second component accounting for 21.66% of the variance resolves the differential response between the four time points. Five clusters were formed in the PCA plot which consisted of all aphid-infested BTx623 (left-down), control BTx623 (right-top), control Tx2783 (left-top), 1- and 3-dpi Tx2783 (right-top), and 6-dpi Tx2783 (right-down). The clustering of similar samples based on the treatments highlights the reproducibility of the experiments. In addition, the PCA results followed and were grouped based on the genotypes and treatments, which indicates that the metabolic and genetic diversity between genotypes is independent of the host response to aphid feeding [28]. The loading plot (red line) indicates that Tx2783 produced higher levels of JA, Ile, ABA, SA, OPDA, and ICA during sorghum–SCA interaction in comparison to BTx623. It also indicates that JA, Ile, and ABA are positively correlated and present in relatively higher levels at 3- and 6-dpi in Tx2783. Similarly, ICA and OPDA are positively correlated and are present in relatively higher amounts at 1- and 3-dpi in Tx2783. In contrast, BTx623 expressed a relatively lower amount of these phytohormones in plants during SCA infestation. The PCA plot also indicated the negative correlation between SA and IAA. These results indicate that during aphid infestation, Tx2783 (resistant) produced relatively higher amounts of phytohormones and the activities of these phytohormones showed an apparent correlation between JA–ABA–Ile and OPDA–ICA.

### 2.4. Differential Expression of Phytohormone Genes

**Figure 5:**
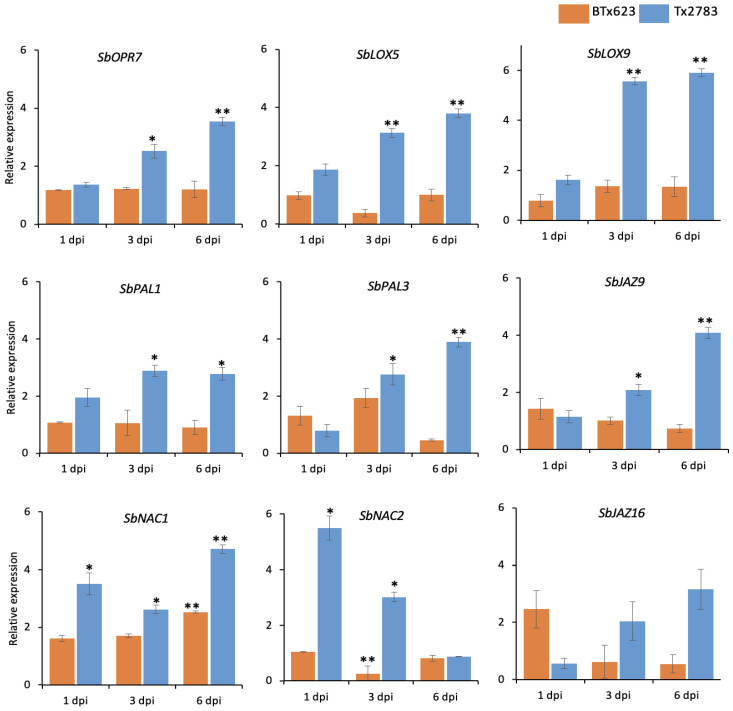
Expression pattern of phytohormone-related genes in response to sugarcane aphid in resistant (Tx2783) and susceptible (BTx623) sorghum genotypes at 1-, 3-, and 6-days post infestation (dpi). qRT-PCR was used to determine the relative expression of each gene using the sorghum *α-tubulin* gene as a control. Error bars in each bar represent the ± standard error (*n* = 3) and asterisks indicate significant differences between the control and aphid treated samples, * *p*  <  0.05, ** *p*  <  0.01. The bars without asterisks are non-significant (*p* > 0.05).

To further support the role of phytohormones in SCA resistance, relative expression of JA-, ABA-, and SA-related marker genes were assessed using the RT-PCR method. The expression pattern of these transcripts were evaluated in both genotypes (Tx2783 and BTx623) after SCA infestation. The expression of *SbLOX9* was significantly higher in Tx2783, around six-fold at 3- and 6-dpi, in comparison to BTx623 (Figure 5). Similarly, *SbLOX9* and *SbOPR7* showed around three- and four-fold increases in Tx2783 at 3- and 6-dpi, respectively. For ABA-related transcripts, *NAC* (*NAM, ATAF1–2*, and *CUC2)* domain proteins, *SbNAC1* and *SbNAC2*, were analyzed in this study. The relative expression of *SbNAC1* in Tx2783 was significantly higher at all time points in comparison to the BTx623 with the highest expression level around five-fold at 6-dpi. Similarly, *SbNAC2* in Tx2783 showed around five-fold and three-fold increases at 1- and 3-dpi, respectively. For SA-related transcripts, two phenylalanine ammonia-lyase (*PAL*) genes, *SbPAL1* and *SbPAL2,* were evaluated in this study. Both *SbPAL1* and *SbPAL2* showed significant upregulation in Tx2783 at 3- and 6-dpi in comparison to BTx623. To investigate the crosstalk between the phytohormones, two *Jasmonate ZIM-domain* (*JAZ*) genes, *SbJAZ9* and *SbJAZ16*, which were previously identified for JA–ABA crosstalk [11], were also evaluated here. It was found that *SbJAZ9* had two-fold and four-fold increases in Tx2783 at 3- and 6-dpi, respectively. Overall, upregulation of the above mentioned phytohormone genes was documented in the resistant line during sugarcane aphid infestation.

### 2.5. Phytohormonal Treatment Induces Resistance in Susceptible Genotypes

**Figure 6:**
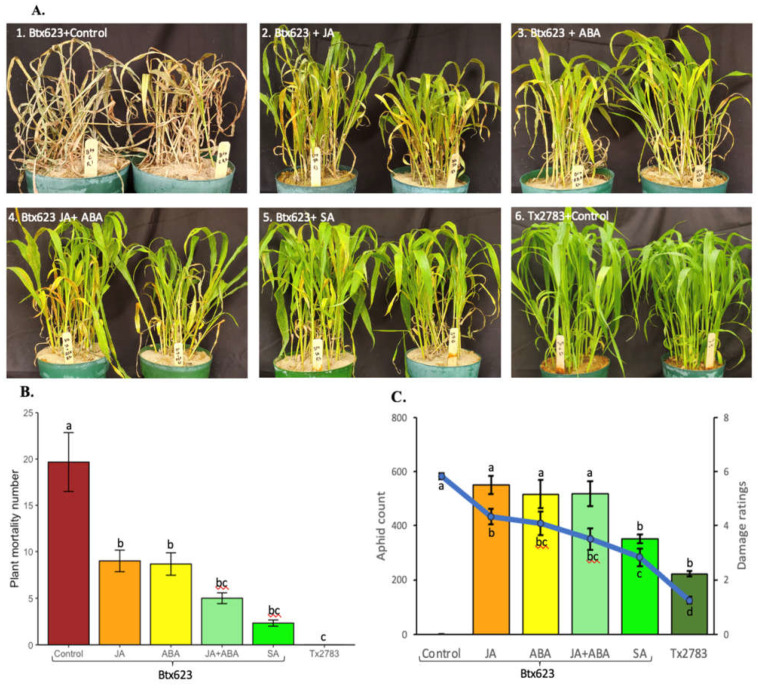
Phytohormone treatment results on BTx623 and Tx2783 after 14 days of SCA infestation. The BTx623 samples were treated with different concentrations of phytohormones and the control samples of BTx623 and Tx2783 were sprayed with distilled water. All treatments were infested with 400 SCA. After 14 days, a status of plant (**A**), plant mortality (**B**) and aphid counts and damage ratings were recorded (**C**) for each treatment. Statistical analysis for plant mortality, aphid counts, and damage ratings was performed through one-way ANOVA and Tukey test. Error bars in plant mortality represent ± standard error (*n* = 3) and in aphid counts and damage ratings represent ± standard error (*n* = 12).

To further validate the role of phytohormones in sorghum defense during SCA attack, a population test was conducted on the seedlings to evaluate the effect of exogenously applied phytohormones. Figure 6A shows the phenotypes of the plants treated with distilled water (control) and different concentrations of phytohormones (JA, ABA, JA + ABA, and SA) and then co-cultivated with SCA after 14-dpi. The phenotype of the sorghum plants with different treatments corresponded to the plant mortality graph (Figure 6B) and aphid count and damage score (Figure 6C). The plant mortality at 14-dpi among various treatments was significantly different (*p*-value = 1.26 × 10^−5^), with the BTx623 control (sprayed with ddH_2_O) showing the highest number, and the Tx2783 control showing 0 dead plants being infested with SCA (Figure 6B). The BTx623 seedlings under two individual treatments with JA and ABA showed a similar number (~10) of dead plants. BTx623 treated with SA showed the lowest number of dead plants (~2) and was on par with BTx623 plants treated with JA + ABA or SCA-infested Tx2783. Similarly, aphid counts and damage scores also showed a highly significant difference (*p*-value = 2 × 10^−16^ and 3.59 × 10^−14^) (Figure 6C). Control BTx623 plants were almost all dead at 14-dpi with damage ratings of 6, so aphid count data could not be recorded for this treatment. The BTx623 plants treated with JA, ABA, and JA + ABA showed similar aphid counts (~500) and damage scores (3.5–4.3) and were significantly at par with one another. Conversely, the BTx623 treated with SA showed significantly lower aphid counts (~351) and damage scores (~2.8). Comparatively, the Tx2783 (resistant) control showed the lowest aphid counts (~223) and damage scores of (~1.25) and was significantly different from all other treatments of BTx623. Overall, 600 µM SA treatment significantly decreased the plant mortality, aphid counts, and damage scores of the BTx623 (susceptible) during SCA infestation.

## 3. Discussion

Phytohormones are key players in mediating plant resistance during pest infestation. To date, very few studies have explored phytohormone levels and their role during aphid infestation in plants. In this study, we focused on the identification of phytohormonal profiles in sorghum plants during SCA infestation and analysis of the role of phytohormones in host-plant defense against aphids for a better understanding of the molecular mechanism underlying plant resistance to SCA.

Two genotypes, resistant (Tx2783) and susceptible (BTx623) to SCA, were selected for a comparative analysis in the experiments. In Table 1, Tx2783 showed a significantly lower aphid population and low damage ratings in comparison to BTx623, which supports the antibiosis and tolerance mechanisms of resistance. Previous studies revealed that Tx2783 carries the resistance to SCA through tolerance and/or antibiosis mechanisms [29,30]. The plant genotypes that carry more than one category of resistance are considered better because tolerance traits limit the yield loss and antibiosis helps to control the population of SCA [29]. Plant resistance is mediated by hormones through various defense signaling pathways [31], which is the initial step in the plant-aphid interaction, leading to activation of resistance genes in plants. The major phytohormones such as JA, SA, ET, ABA, and GA are important players in defense signaling pathways. These hormones can act individually or together, with antagonistic or synergistic interactions, in the plant signal network [32].

JA, JA-Ile, and their precursor OPDA play central roles in the plant response to herbivore attack [33]. The increased level of endogenous JA in plants after wounding correlates with the induced defense response and vice-versa [32]. The amounts of JA and JA-Ile in the aphid-infested resistant genotype (Tx2783) were significantly increased in comparison to the control samples (Figure 3). Similarly, the levels of JA and OPDA were significantly increased in the aphid-infested resistant genotype in comparison to the susceptible genotype (BTx623). Similar results of high levels of JA and JA-Ile were noted in tolerant *Glycine max* during *Aphis glycines* infestation [34]. The high level of OPDA was reported in tolerant sorghum during SCA infestation [35]. A two-week picture of the upregulation of JA and JA-Ile in plants in response to aphid feeding can be seen with the combination of our study during early infestation stages (1-, 3-, and 6-dpi) and the study of Grover et al. [35] during 7- and 14-dpi, which showed similar trends of elevated phytohormone levels. The increased level of JA was supported by the upregulation of the respective marker genes. The marker genes related to JA, *SbLOX5*, *SbLOX9*, and *SbOPR7,* were significantly induced in the resistant genotype. Previous research has also shown that the *LOX* genes are highly induced in plants during aphid infestation that exhibited antibiosis [31]. *LOX* is the key enzyme in the JA biosynthesis pathway and has been studied in response to aphid feeding among many plant species such as *Solanum lycopersicum* [36], Arabidopsis [37], sorghum [10], and *Zea mays* [38]. The increase of JA during herbivory induced the production of the defensive compound indole glucosinolates (GS), which plays defensive and protective roles against insect pests [19]. The OPDA, a precursor of JA, can act independently during defense by promoting plant growth and development in tolerant sorghum genotypes [35]. Chapman et al. [34] demonstrated that JA and ABA contributed to *G. max* tolerance against *A. glycines*. Taken together, the results suggest that highly induced JA and OPDA in plants produce defense molecules and promotes growth, making sorghum tolerant to sugarcane aphid.

SA, an important phytohormone, generates a wide range of metabolic and physiological responses to biotic stress in plants, and provides resistance against various plant pathogens and insects, including phloem-feeding pests [39,40]. The results from Figure 3 revealed significantly higher levels of SA in the aphid-infested resistant genotype, which was further supported by upregulated expression of *SbPAL* genes. The *PAL* genes involvement in SA biosynthesis is well established and induced *PAL* expression leads to SA accumulation in several plants [41,42]. Upregulation of *PAL* expression has been reported in various plants in response to pest/pathogen infection. For example, *PAL* expression was increased in sorghum against SCA infestation according to unpublished data, and in *Medicago truncatula* against *Acyrthosiphon kondoi* [31]. Similarly, the induction of SA has been reported in *T. aestivum* against *Diuraphis noxia* [43] and in *Horedum vulgare* against *Schizaphis graminum* [44]. During herbivory, induced SA produces phenolic compounds and flavonoids which enhance defense resistance in plants [45]. Several researchers have shown the activation of the SA pathway as a general mechanism of antibiosis or antixenosis resistance in resistant host plants [32]. Research in *Brassica napus* found SA plays a role in antibiosis resistance against *Brevicoryne brassicae* [45]. Based on the above-mentioned evidence, we suggest that SA was induced in the resistant sorghum during the sorghum and SCA interaction, subsequently activating resistance genes to protect the host plants from aphid attack.

The ABA role in plant abiotic stress and pathogen resistance is well documented, while its specific role in plant-pest interactions is less studied. Flors et al. [46] revealed the important role of ABA during plant-insect interactions. The level of ABA was significantly upregulated only at 6-dpi (Figure 3) of Tx2783 in comparison to other time points, while ABA-related transcripts (*SbNAC1* and *SbNAC2*) were significantly increased at all time points (Figure 5). Similar results of higher expression of ABA and *NAC* genes were reported in tolerant *G. max* against *A. glycines* [34]. The high upregulation of ABA-related genes was also reported in resistant sorghum against greenbug aphid, *Schizaphis graminum* [9,47]. Furthermore, ABA, when produced in combination with JA, acts synergistically on the expression of the defense genes during insect attack and wounding [26,48]. The PCA plot (Figure 4) also supports the correlation between JA and ABA during sorghum plant and SCA interaction, which suggests a crosstalk between JA and ABA in plants and is possibly mediated by the *JAZ* genes [26,48]. Evidently, *SbJAZ16* and *SbJAZ5* were reported as the mediator during crosstalk between JA and ABA in sorghum plants [11]. The significant expression of *SbJAZ9* in Tx2783 during SCA infestation suggests the involvement of this gene functionally in JA–ABA crosstalk. Thus, we suggest the role of ABA in plant defense against aphids through crosstalk between JA and ABA to activate resistance genes in resistant plants.

Auxins (IAA and ICA) regulate plant growth and development and are negatively correlated with SA-mediated plant defenses [49,50]. The negative correlation between IAA and SA was exhibited in our PCA plot (Figure 4). The negative correlation during herbivory is a trade-off of growth during the defense response. The level of IAA and ICA was significantly higher in Tx2783 at 1- and 3-dpi, respectively. A higher level of auxins was also reported in *Nicotiana attenuata* against *Manduca sexta* [51]. An increased level of auxin is related to lignification of the cell wall to increase leaf rigidity and stem strength to limit herbivory attack [52]. Based on these, we suggest that resistant plants produce auxin to maintain plant growth and development to help the plant tolerate aphid infestation, thus helping the plant avoid or reduce damage from aphid feeding.

The results of phytohormone treatment to the susceptible sorghum genotype indicate the role of SA and JA + ABA in SCA resistance. The SA and JA + ABA treatment significantly decreased plant mortality, aphid numbers, and damage scores of the susceptible genotype during SCA infestation (Figure 6). The hormone SA generally induces plant defenses against biotrophic pathogens [53]. A previous study showed that *Brassica napus* treated with SA decreased the *Brevicoryne brassicae* population and induced antibiosis resistance [45]. Similarly, exogenous application of SA enhanced the resistance of *Oryza sativa* to *Nilparvata lugens* [54]. The activation of the SA pathway in plants is believed to be a general mechanism of antibiosis or antixenosis in resistant host plants [32]. Similarly, a susceptible sorghum genotype treated with 100 uM JA, 50 uM ABA, or combination of JA + ABA showed significantly lower numbers of plant mortality in comparison to the control (Figure 6). Among those, the combined treatment of JA + ABA had a significantly lower number of plant mortality. Chapman et al. [34] demonstrated that JA and ABA contributed to soybean (*Glycine. max*) tolerance against *Aphis glycines*. Evidently, our results generated from this study in sorghum are consistent with previous reports in other plant species and concludes that SA and the combination of JA + ABA can induce the resistance in sorghum against SCA, and further suggests antibiosis (SA) and tolerance (JA + ABA) mechanism of resistance working in sorghum plants.

## 4. Materials and Methods

### 4.1. Plant Materials and Sugarcane Aphids

Sugarcane aphid (SCA, *Melanaphis sacchari* Zehntner) cultures were established from a single parthenogenetic female. Sugarcane aphid colonies were reared and maintained on susceptible sorghum plants (Tx7000) in the greenhouse at USDA-ARS Plant Science Research Laboratory, Stillwater, Oklahoma; thus, fresh aphid colonies were always available when plants were ready for infestation. Two sorghum genotypes, BTx623 (susceptible) and Tx2783 (resistant), were selected as parallel lines for this study. Sorghum seeds from Tx2783 and BTx623 were grown in the greenhouse at constant temperature (28 ± 2 °C) and 60% relative humidity under constant photoperiod of 14 h-light/10 h-dark. When sorghum seedlings reached the 2–3 leaf stage (8–10 days from sowing), they were infested with 20 apterous sugarcane aphid adults to the adaxial surface of the first true leaf. Each infested plant and control plants (not infested with aphids) were covered with a transparent cylindrical cage with nylon mesh on the top. To confirm the differential responses of the two genotypes to aphid infestation, the aphid count was recorded at 1-, 3-, 6-, 9-, and 12-dpi from ten independent plants of each infested lines. In addition, plant damage scores were recorded using a scale of 0 to 6, with 0 being no damage, 1 damage < 20%, 2 damage 21–40%, 3 damage 41–60%, 4 damage 61–80%, 5 damage >  80%, and 6 being a dead plant. To evaluate the differential response of two genotypes, samples (a whole seedling above ground) were collected from the two genotypes infested with sugarcane aphids and without (control) at 0-, 1-, 3-, and 6- dpi. Each sample harvested had three biological replicates for each time point and they were frozen immediately in liquid nitrogen and stored at −80 °C. The control samples were collected at each time point to eliminate the circadian rhythm effect on gene expression.

### 4.2. Sample Preparation, Phytohormone Extraction and Quantification

For chemical analysis, sorghum seedling samples were prepared according to the published protocol [55]. Briefly, after freeze-drying, lyophilized sorghum tissue was homogenized in 5 mL polypropylene tubes using stainless steel beads and the Bullet Blender^®^ Storm5 homogenizer (Next Advance, Averill Park, NY, USA). All homogenized leaf tissues for each sample were combined and mixed thoroughly. Three analytical replicates were prepared by adding a 19–21-mg portion of the lyophilized leaf tissue to a 2-mL glass vial. Phytohormones were extracted by adding 1 mL of methyl-tert-butyl-ether (MTBE) solution containing 6:3:1 MTBE:methanol:water (*v*/*v*/*v*) and vortexing at 4 °C for 60 min. Next, glass vials were centrifuged for 15 min at 3500× *g*. A 400-μL aliquot of the extraction was transferred to a fresh 2-mL glass vial, dried under nitrogen, re-suspended in 100 μL of methanol, and then stored at −80 °C until LC-MS analysis.

### 4.3. Quantification of Phytohormones by LC-MS/Phytohormone Quantification

For phytohormone quantification, the following important hormone classes including jasmonic acid (JA), salicylic acid (SA), abscisic acid (ABA), indole-3-carboxylic acid (ICA), indole-3-acetic acid (IAA), 12-oxo-phytodienoic acid (OPDA), and jasmonyl-L-isoleucine (JA-Ile) were measured in the seedling samples of BTx623 and Tx2783 at 0-, 1-, 3-, and 6- dpi, respectively. Liquid chromatography–mass spectrometry (LC-MS) assay was performed at the Metabolomics Core Facility of University of California at Riverside using the protocol as previously described [55].

### 4.4. RNA Extraction and Quantitative Real-Time PCR Analysis

A Trizol reagent (Invitrogen, Carlsbad, CA, USA) was used to extract the total RNA from 100mg of each sample and then was treated with DNase (Turbo DNA-free kit, Thermo Fisher Scientific, Waltham, MA, USA). A total of 2.5 μg of RNA was reverse-transcribed using the GoScript reverse transcriptase kit (Promega, Madison, WI, USA) and the resulted cDNA was diluted four-fold before using for the qRT-PCR reaction. For RT-PCR, the genes responsive to JA, SA, and ABA were selected through a literature search and alignment study. The primers for the genes were designed using the IDT DNA program (https://www.idtdna.com/PrimerQuest/Home/ accessed on 12 August 2021), which are listed in Table S1. A sorghum *α-Tubulin* gene (*Sobic.001G107200*) was used as the internal control as described previously [11,56]. qRT-PCR was performed on a Bio-Rad iCycler thermal cycler (Bio-Rad Laboratories, Inc., Hercules, CA, USA) using the iTaq™ universal SYBR^®^ green supermix (Bio-Rad Laboratories, Inc.). The qRT-PCR reaction was performed in a volume of 10 μL, containing 1 μL of cDNA, 0.4 μL (10 μM) each of the reverse and forward primers, 5 μL of SYBR green master mix, and 3.2 μL of ddH2O under the following conditions: one cycle at 95 °C for 3 m, 40 cycles at 95 °C for 10 s and 55 °C for 30 s, followed by one cycle each of one min at 95 °C and 55 °C. The final melting curve was 81 cycles at 55 °C for 30 s.

### 4.5. Analysis of Phytohormonal Effect on Host Plant Resistance to SCA

To further confirm the role of SA, JA, and ABA in response to the SCA infestation, a small population test was performed. Around 20–25 plants of two genotypes (BTx623 and Tx2783) were planted in a pot. The 2–3 leaf stage (8–10 days) seedlings were sprayed with 600 µM SA [57], 100 µM MeJA [58], 50 µM ABA [59], and sterile distilled water (ddH_2_O, control) to each pot separately. The phytohormones (JA and SA) were mixed separately in distilled water and stirred until dissolved completely and 0.1% of Tween20 was added to each solution. For preparing ABA solution, 39.63 mg of ABA was dissolved in 1ml of ethanol and then diluted in 3 L of distilled water to make a desired concentration (50 uM of ABA). A total of 3 mL of Tween20 (0.1%) was added to this solution and mixed properly. The phytohormones solutions (SA, JA, ABA, and JA + ABA) and control (ddH_2_O) were sprayed separately to each pot until the plants were soaked completely with the solution. Each phytohormone treatment and control had three replicates. After spraying, the pots were covered with a transparent cylindrical cage with nylon mesh on the top. After 6 h, each sprayed pot was infested with 400 SCA. At 14-dpi, plant mortality rate was recorded from each pot. In addition, aphid numbers and plant damage scores were recorded from four random plants from each pot.

### 4.6. Statistical Analysis

A principal component analysis (PCA) was carried out to explore the statistical correlations between phytohormones produced in the resistant and susceptible sorghum genotype and control samples using the PCA package in R software. For the aphid count data and phytohormone quantification graphs, the *t*-test was used to estimate the significant difference between the two genotypes. Similarly, for the phytohormones quantification graph, one-way ANOVA and Tukey test was used to find the significant difference between treatments (0, 1-, 3-, and 6-dpi) of the same genotype. The relative expression level of each gene was calculated using the 2−ΔΔCt method and the data presented are the averages of three biological and two technical replicates. For the expression analysis, *t*-test was used to estimate the significant differences between SCA-infested and control samples (* *p* < 0.05 and ** *p* < 0.01). The phytohormone treatment results of plant mortality, aphid counts, and damage ratings were analyzed through one-way ANOVA and Tukey test.

## 5. Conclusions

In summary, this report presents a comparative analysis of two sorghum genotypes Tx2783 and BTx623 in parallel, which reveal that the virulent sugarcane aphid (SCA) can initially establish feeding on seedlings of both lines, but only Tx2783 proved to be resistant and was able to defend against SCA. Resistant plants have a variety of built-in mechanisms to prevent unexpected attack, including a sophisticated molecular defense system such as phytohormone-mediated defense. The expression profiles of phytohormones in aphid-infested plants was assessed using the metabolomic approach. We demonstrated that seven important phytohormones were differentially expressed between the resistant and susceptible plants and at various time points during plant-aphid interaction. These results confirmed the important role of phytohormones, JA, SA, ABA, and Auxin, in host-plant defense against sugarcane aphids, and these hormones interact together as a defense signaling network to fine-tune the defense. This research also provides important insight into the synergistic relationship between JA and ABA and antagonistic relationship between SA and IAA, which are regarded as important cross talkers in the defense signaling pathway. To gain a more complete understanding of the molecular mechanism of host-plant resistance to SCA, a correlation of differential phytohormone expression and resistance gene action timeline needs to be analyzed in a future study. Additionally, chemical screening of aphid-induced metabolites in the host plant will help better describe phytohormone-mediated aphid resistance in plants.

## Figures and Tables

**Table 1:** Aphid count and damage ratings data for two sorghum genotypes subjected to SCA infestation.

dpi	Aphid Count			Damage Ratings	
	Btx623	Tx2783	*p*-Value	Btx623	Tx2783
1	24 ± 2.23	14 ± 1.11	***	0	0
3	41 ± 4.23	26 ± 2.79	**	1	0
6	192 ± 21.1	65 ± 4.66	***	1	0
9	542 ± 41.6	153 ± 9.80	***	2	1
12	913 ± 54.3	309± 21.8	***	4	2
15	-	-	-	6	3

The aphid count in each genotype is the mean of ten plant samples ± standard error. The asterisk (*) at the *p*-value represents the significant difference between the genotype at the same time points (*** *p* < 0.001, ** *p* < 0.01; *t*-test).

## Data Availability

Not applicable.

## Supplementary Materials

The following supporting information can be downloaded at: https://www.mdpi.com/article/10.3390/ijms232213782/s1, Table S1. Primers used for the RT-PCR analysis.
