# Supplementary material for: Revealing Differential Expression of Phytohormones in Sorghum in Response to Aphid Attack Using the Metabolomics Approach
Source: Int J Mol Sci. 2022 Nov 9;23(22):13782. doi: 10.3390/ijms232213782 (PMC9699302; doi:10.3390/ijms232213782)
Supplement: Supplementary file 1 [file ijms-23-13782-s001.zip › ijms-1956594-supplementary.pdf]

Revealing differential expression of phytohormones in sorghum in response to aphid attack using the metabolomics approach

Table S1. Primers used for the RT-PCR analysis.

Name	Gene ID	Forward	Reverse
SbOPR7	Sobic.007G151100	CAACTATGGCATGTAGGGAGAG	GATCCATCGGGCATCAGTATC
SbNAC1	Sobic.001G515800	CTGGGTCATGAACGAGTACC	CTCTGTTCCAGCTCCTTCAG
SbNAC2	Sobic.005G018500	ACCCTCCTCCTCTAATCTACAC	GCGTTTGTGTGTGGCAAATA
SbPAL1	Sobic.004G220300	GCCCTCCGTGTTCTCCAAGATCAC	TTCGCCACGGGGGCGGTGCCCTC
SbPAL3	Sobic.004G220500	GGTGCTCGTTGCCATCA	TCTCTCTCTCTATCCGCTTCTC
SbLOX5	Sobic.006G095600	GTTCTTCATCGAGAGCATCGT	GTAGGGCTTGTTGGTGAAGA
SbLOX9	Sobic.004G078600	TGGAGGAGCTGAGGAAGAA	GTCGTTGTACACGTCGTAGTC
SbJAZ9	Sobic.001G482700	TCAAGAGGTTCCTCGAGAAGAG	TCCTTAACAGCAGGAGGCT
SbJAZ16	Sobic.006G056400	GCCACTCACAAGAACCAAATC	GTTCCTCCCGATCCCATTATTC
α-Tubulin	Sobic.001G107200	GAGGTGACGATGCTTTCAACAC	CACAGGTCAACAATCTCCTTGC
